# Nonalcoholic fatty liver disease and type 2 diabetes: an observational and Mendelian randomization study

**DOI:** 10.3389/fendo.2023.1156381

**Published:** 2023-05-08

**Authors:** Yuetian Yu, Yuefeng Yu, Yuying Wang, Yi Chen, Ningjian Wang, Bin Wang, Yingli Lu

**Affiliations:** Institute and Department of Endocrinology and Metabolism, Shanghai Ninth People’s Hospital, Shanghai JiaoTong University School of Medicine, Shanghai, China

**Keywords:** nonalcoholic fatty liver disease, type 2 diabetes, Mendelian randomization, China, UK Biobank

## Abstract

**Introduction:**

Nonalcoholic fatty liver disease (NAFLD) and type 2 diabetes mellitus (T2DM) are both chronic multisystem diseases that cause tremendous health burdens worldwide. Previous epidemiological studies have found a bidirectional relationship between these two diseases; however, their causality remains largely unknown. We aim to examine the causal relationship between NAFLD and T2DM.

**Methods:**

The observational analysis included 2,099 participants from the SPECT-China study and 502,414 participants from the UK Biobank. Logistic regression and Cox regression models were used to examine the bidirectional association between NAFLD and T2DM. Two-sample Mendelian randomization (MR) analyses were conducted to investigate the causal effects of the two diseases using summary statistics of genome-wide association studies from the UK Biobank for T2DM and the FinnGen study for NAFLD.

**Results:**

During the follow-up, 129 T2DM cases and 263 NAFLD cases were observed in the SPECT-China study, and 30,274 T2DM cases and 4,896 NAFLD cases occurred in the UK Biobank cohort. Baseline NAFLD was associated with an increased risk of incident T2DM in both studies (SPECT-China: OR: 1.74 (95% confidence interval (CI): 1.12–2.70); UK Biobank: HR: 2.16 (95% CI: 1.82–2.56)), while baseline T2DM was associated with incident NAFLD in the UK Biobank study only (HR: 1.58). Bidirectional MR analysis showed that genetically determined NAFLD was significantly associated with an increased risk of T2DM (OR: 1.003 (95% CI: 1.002–1.004, *p<* 0.001)); however, there was no evidence of an association between genetically determined T2DM and NAFLD (OR: 28.1 (95% CI: 0.7–1,143.0)).

**Conclusions:**

Our study suggested the causal effect of NAFLD on T2DM development. The lack of a causal association between T2DM and NAFLD warrants further verification.

## Introduction

Nonalcoholic fatty liver disease (NAFLD) is defined as an abnormal accumulation of fat in the liver without significant alcohol intake (1). With a global estimated prevalence of 25%, it is the most prominent cause of liver disease worldwide (2). Due to its high potential in developing liver fibrosis, liver cirrhosis, and hepatocellular carcinoma, NAFLD has already caused considerable clinical and health burdens worldwide (3, 4).

NAFLD is a multisystem disease that can affect extrahepatic organs and regulatory pathways, causing other chronic diseases and related complications (5). Over the past decade, compelling observational studies have demonstrated that NAFLD and type 2 diabetes mellitus (T2DM) are two pathologic conditions that frequently coexist, and there seems to be a bidirectional relationship between them (6). The presence of NAFLD substantially increases the risk of incident T2DM, and such risk parallels the severity of NAFLD (7–9). However, the studies targeting baseline NAFLD and incident T2DM have been mostly conducted in the Asian population, and relevant evidence in the Western population is lacking (10). On the other hand, the prevalence of NAFLD is more than twofold higher in patients with T2DM than in the general population. This association remains similar among different races, but the strength of the association seems to be stronger in white Europeans than in Asians (11).

Although the bidirectional relationship between NAFLD and T2DM has been widely reported, the causality remains largely uncertain due to potential confounding factors or the reverse causation bias within observational studies (12). As a result, it is crucial to dissect the causal relationship between NAFLD and T2DM to better understand the disease etiology and inform effective diagnostic, therapeutic, and preventive strategies.

In recent years, Mendelian randomization (MR) analysis, a form of instrumental variable (IV) analysis, has profoundly equipped researchers with tools for estimating causal inference between exposures and outcomes (13). This approach carries two merits of minimizing confounding and diminishing reverse causality because genetic variants are randomly allocated at conception (thus unrelated to self-adopted and environmental factors) and cannot be modified by the development and progression of the disease. Bidirectional MR is an extension of traditional MR in which the exposure–outcome causal relationship was explored from both sides, providing an efficient way to ascertain the direction of a causal relationship (14).

In this study, we first examined the observational association between T2DM and NAFLD in both the Chinese and White populations. We then investigated the direction of the causal relationship using the bidirectional MR method based on online genome-wide association study (GWAS) data.

## Methods

### Study population

SPECT-China (registration number ChiCTR-ECS-14005052; www.chictr.org.cn) is a population-based study investigating the prevalence of metabolic diseases and risk factors in East China. A stratified cluster sampling method was used to select a sample in the general population at 23 sites across Shanghai, Zhejiang, Jiangsu, Anhui, and Jiangxi Province. The sampling process was stratified according to rural/urban areas and economic development. Chinese citizens aged 18 years old and above who have lived in their current residence for 6 months or longer were selected for our study. After excluding those with acute illness, severe communication problems, or unwilling to participate, a total of 6,899 subjects were included in the SPECT-China study from February to June 2014 (15).

Between January and June 2019, the participants were invited to attend a first-round follow-up visit. A total of 2,171 participants attended the follow-up survey. We then excluded 72 participants with missing liver ultrasound results at baseline or in the follow-up, leaving 2,099 eligible participants for further analysis (**Supplementary Figure S1**).

The study protocol was approved by the Ethics Committee of Shanghai Ninth People’s Hospital, Shanghai Jiao Tong University School of Medicine (approval number 2013 (86)). All procedures followed were in accordance with the ethical standards of the responsible committee on human experimentation (institutional and national) and with the Helsinki Declaration. Informed consent was obtained from all participants in the study.

The UK Biobank (UKB) is a population-based prospective cohort study, including more than 500,000 community-dwelling adults aged 37–73 years across the UK between 2006 and 2010 (https://www.ukbiobank.ac.uk/). We declare that all data are publicly available in the UKB repository (16). The UKB received ethical approval from the UK National Health Service, the National Research Ethics Service North West, the National Information Governance Board for Health and Social Care in England and Wales, and the Community Health Index Advisory Group in Scotland. All participants provided written, informed consent. This study was approved by the UK Biobank (application number 77740).

### Data collection and measurements

Sociodemographic characteristics, medical history, family history, and lifestyle factors were obtained through our questionnaire. Regional economic status was assessed by the gross domestic product per capita at each site and categorized into high and low economic status according to the national level in 2013 ($6,807 per capita from the World Bank) (17). Well-trained and experienced staff conducted anthropometric measurements according to a standard protocol at each study site, providing us with weight, height, waist circumference, and blood pressure. Venous blood samples were drawn after overnight fasting for at least 8 h. These samples were immediately stored at −20°C and sent to the central laboratory by air on dry ice within 4 h.

### Instrumental variables

First, we extracted independent SNPs for diabetes from the summary statistics of GWAS, which were publicly downloadable from the IEU OPEN GWAS PROJECT (https://gwas.mrcieu.ac.uk/), with the same Batch ID of “ukb-b.” We then explored the FinnGen dataset, which had no overlapped participants with the dataset mentioned before, for GWAS summary statistics of NAFLD. The FinnGen Biobank GWAS was performed by the FinnGen team (https://r4.finngen.fi/) and is available on the IEU OPEN GWAS PROJECT. Finally, we pruned the genetic variants within a 250-kb window to include independent SNPs (r2< 0.1). All the SNPs used in the study are shown in **Supplementary Table S1**.

### Definition of variables

In the SPECT-China dataset, current smoking was defined as having smoked at least 100 cigarettes in one’s lifetime and currently smoking cigarettes. T2DM was determined by fasting plasma glucose at ≥7.0 mmol/l and/or HbA1c ≥48 mmol/mol (6.5%) and/or a self-reported previous diagnosis by healthcare professionals according to the 2010 ADA criteria (18). Liver fat accumulation (steatosis) was detected by ultrasound; the presentation of steatosis included increased liver echogenicity, stronger echoes in the hepatic parenchyma as compared with the renal parenchyma, vessel blurring, and narrowing of the lumen of the hepatic veins according to the criteria of Saadeh et al. (19). NAFLD was defined as ultrasound evidence of fatty liver and the exclusion of secondary causes (having a history of excessive consumption (30 g/day in men and 20 g/day in women) of pure alcohol, self-reported viral hepatitis, and using medications associated with secondary NAFLD (corticosteroids, amiodarone) (20).

In the UK Biobank dataset, T2DM and NAFLD were ascertained using linkage with hospital inpatient records. The date and cause of hospital admissions were obtained from record linkage to Health Episode Statistics (England and Wales) and the Scottish Morbidity Records (Scotland). We defined outcomes according to the International Classification of Diseases, edition 10 (ICD-10): E11 for T2DM and K76.0 for NAFLD after the exclusion of viral hepatitis (B15–B19).

We alternatively used the fatty liver index (FLI), a noninvasive algorithm for identifying liver steatosis at baseline. The formula of FLI was as follows:


$$FLI=\frac{\left(e^{0.953*loge(triglycerides)+0.139*BMI+0.718*loge(ggt)+0.053*waistcircumference-15.745}\right)}{\left(1+e^{0.9533*loge(triglycerides)+0.139*BMI+0.718*loge(ggt)+0.053*waistcircumference-15.745}\right)}*100$$

I was expressed as a value ranging from 0 to 100, and previous studies have validated that it matched the observed percentages of patients with hepatic steatosis accurately (21). NAFLD was based on an FLI ≥ 60 and the exclusion of viral hepatitis, excessive alcohol consumption (alcohol consumption: ≥ 30 g/day for male participants and 20 g/day for female participants), or aspartate transaminase or alanine aminotransferase > 500 U/L (11).

### Statistical analysis

Continuous variables were expressed as mean (standard deviation), and categorical variables were described as percentages (%). Characteristics of the study sample were compared by the *t*-test for continuous variables and Pearson’s Chi-square test for categorical variables. The multivariate Cox regression model was used in the UK Biobank dataset, and the logistic regression model was used in the SPECT-China dataset to evaluate the association between NAFLD and diabetes. The follow-up time was calculated from the baseline date to the diagnosis of outcome, death, or the censoring date (30 May 2022), whichever came first. The model was adjusted for age, gender, education level, living area, smoking status, drinking status, economic status, and BMI. Family history of diabetes was additionally adjusted when assessing the association between baseline NAFLD and incident T2DM.

For the two-sample MR method, we mainly performed an inverse-variance–weighted (IVW) MR analysis to verify the causal association between NAFLD and diabetes. Moreover, we applied MR-Egger regression and the weighted median approach. If there was no evidence of directional pleiotropy (*p*-value for MR-Egger intercept > 0.05), the estimate from the IVW method was considered the most reliable indicator.

A two-tailed *p*< 0.05 was considered statistically significant. All analyses were performed using R version 4.2.0 and SPSS software version 26.0.

## Results

### Baseline characteristics

**Table 1** shows the baseline characteristics of the participants. In the SPECT-China dataset, a total of 2,099 participants (mean age ± SD: 53.54 ± 11.35 years) were included in the final analyses. Participants who developed diabetes were relatively older, richer, had a lower education level, and tend to have a habit of drinking. They also had a higher level of BMI, were more likely to have a family history of diabetes, and had a higher prevalence of NAFLD. Meanwhile, compared with those who did not develop NAFLD during follow-up, participants with incident NAFLD were more likely to be women, have a habit of drinking and smoking, have a higher economic status, live in urban areas, and have a higher level of BMI. Further analysis of the UK Biobank dataset showed similar results (**Table 1**; **Supplementary Table S2**).

**Table 1:** Baseline characteristics of the study population.

	Participants without baseline T2DM		Participants without baseline NAFLD	
	Incident T2DM	No incident T2DM	Incident NAFLD	No incident NAFLD
SPECT-China				
Number of participants	129	1,707	263	882
Age at recruitment (years)	57.67 ± 8.60^*^	52.38 ± 11.52	52.90 ± 10.92	52.03 ± 10.92
Gender (%)				
Women	58.1%	58.3%	42.6%^*^	35.8%
Men	41.9%	41.7%	57.4%^*^	64.2%
Current smoking (%)	29.5%^*^	19.3%	24.3%^*^	17.6%
Current drinking (%)	31%	32%	38.1%^*^	29.3%
Economic status (%)				
High	89.9%^*^	80%	90.9%^*^	77.7%
Low	10.1%^*^	20%	9.1%^*^	22.3%
Living area (%)				
Rural	65.9%	61.2%	74.5%^*^	64.7%
Urban	34.1%	38.8%	25.5%^*^	35.3%
College education or above (%)	10.1%^*^	22.1%	16.7%	19.2%
BMI (kg/m^2^)	26.43 ± 3.45^*^	23.81 ± 3.23	24.27 ± 2.96^*^	22.30 ± 2.66
Family history of diabetes (%)	15.5%^*^	9.8%		
Baseline NAFLD (%)	43.4%^*^	19.2%		
Baseline T2DM (%)			8.4%	6.5%
UK Biobank				
Number of participants	30,274	458,610	4,896	496,867
Age at recruitment (year)	59.2 ± 7.3^*^	56.2 ± 8.1	56.8 ± 7.9^*^	56.5 ± 8.1
Gender (%)				
Women	41.8^*^	55.7	53.1^*^	54.4
Men	58.2^*^	44.3	46.9^*^	45.6
Smoking status (%)				
Never	45.3^*^	55.7	45.9^*^	54.9
Previous	40.8^*^	33.9	39.4^*^	34.6
Current	13.9^*^	10.4	14.7^*^	10.5
Drinking status (%)				
Never	8.1^*^	4.1	6.7^*^	4.4
Previous	6.1^*^	3.3	7.0^*^	3.6
Current	85.8^*^	92.6	86.3^*^	92.0
Townsend deprivation index	−0.4 ± 3.4^*^	−1.4 ± 3.0	−0.4 ± 3.4^*^	−1.3 ± 3.1
Living area (%)				
Rural	11.2^*^	15.0	12.1^*^	14.7
Urban	88.8^*^	85.0	87.9^*^	85.3
College Education or above (%)	21.0^*^	33.1	21.4^*^	32.2
BMI (kg/m^2^)	31.4 ± 5.6^*^	27.0 ± 4.5	31.3 ± 5.7^*^	27.4 ± 4.8
Family history of diabetes (%)	24.6^*^	13.5		
Baseline NAFLD (%)	0.4^*^	0.1		
Baseline T2DM			8.1^*^	2.6

Continuous variables were expressed as mean ± SD, and categorical variables were described as a percentage (%). Characteristics of the study sample were compared by the t-test for continuous variables and Pearson’s Chi-square test for categorical variables.

^*^p< 0.05, significantly different from that in the nonprogressor group.

NAFLD, nonalcoholic fatty liver disease; T2DM, type 2 diabetes mellitus.

### Association between diabetes and NAFLD

**Figure 1:**
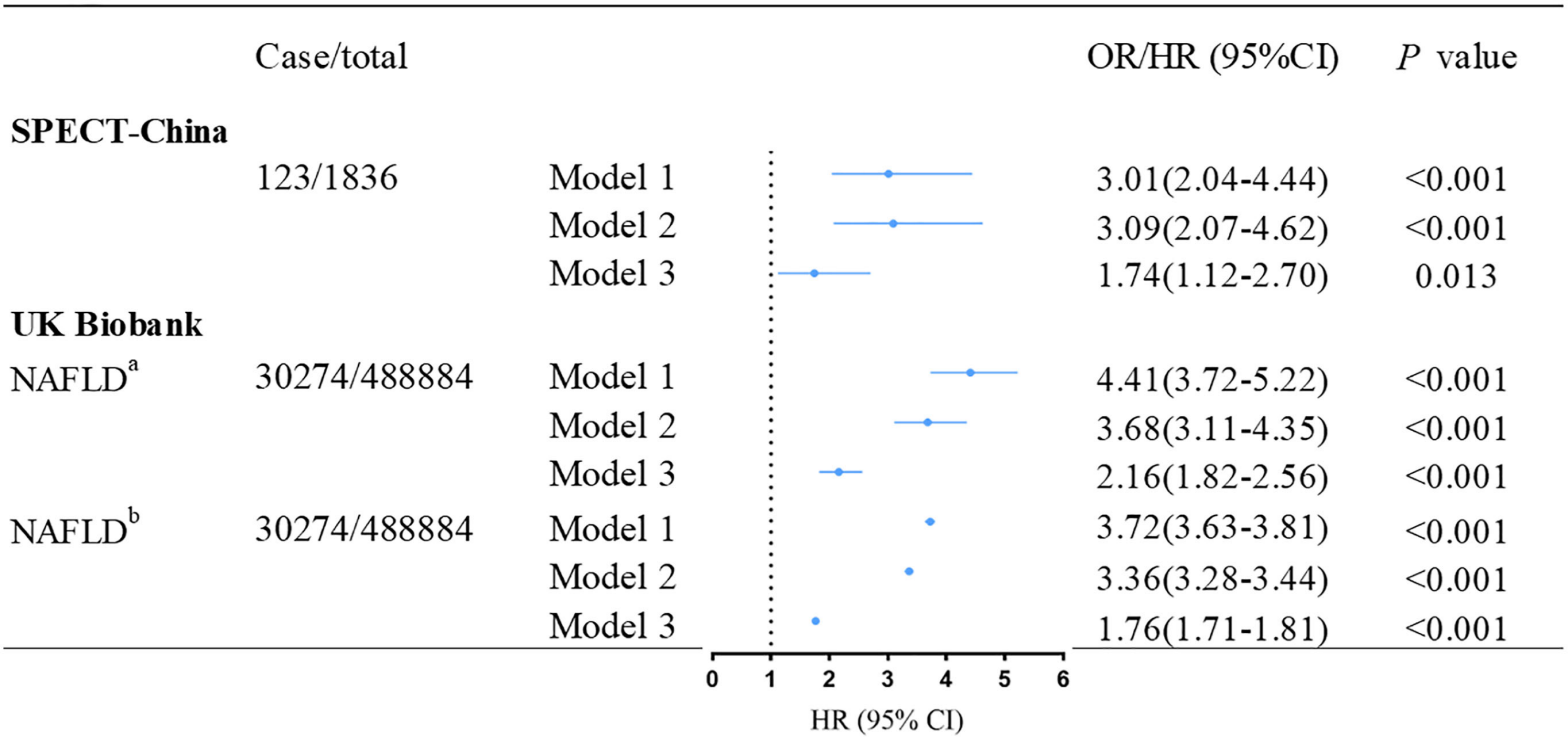
Association between baseline NAFLD and incident T2DM in SPECT-China and UK Biobank. ^a^NAFLD was ascertained according to hospital inpatient records. ^b^NAFLD was defined by fatty liver index. Model 1 was adjusted for age, and gender. Model 2 was further adjusted for education level, living area, smoking status, drinking status and economic status. Model 3 was additionally adjusted for BMI and family history of diabetes based on Model 2. OR, odd ratio; HR, hazard ratio; CI, confidence interval; NAFLD, nonalcoholic fatty liver disease; T2DM, type 2 diabetes mellitus.

**Figure 1** demonstrates the association between baseline NAFLD and incident T2DM. After adjusting for age, gender, education level, living area, smoking status, drinking status, economic status, and BMI, we found that baseline NAFLD was associated with a significantly higher risk of incident T2DM both in the SPECT-China dataset (OR: 1.74 (95% confidence interval (CI):1.12–2.70); *p* = 0.013) and the UK Biobank dataset (HR: 2.16 (95% CI: 1.82–2.56); *p<* 0.001). This result remained unchanged when we additionally used FLI to define NAFLD (HR: 1.76 (95% CI: 1.71–1.81); *p<* 0.001).

**Figure 2:**
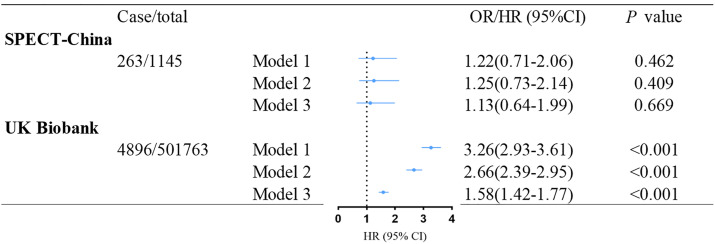
Association between baseline T2DM and incident NAFLD in SPECT-China and the UK Biobank. Model 1 was adjusted for age and gender. Model 2 was further adjusted for education level, living area, smoking status, drinking status, and economic status. Model 3 was additionally adjusted for BMI based on model 2. OR, odd ratio; HR, hazard ratio; CI, confidence interval; NAFLD, nonalcoholic fatty liver disease; T2DM, type 2 diabetes mellitus.

**Figure 2** shows the association between baseline T2DM and incident NAFLD. After multivariable adjustment, we observed that baseline T2DM is significantly associated with incident NAFLD (HR: 1.58 (95% CI: 1.42–1.77); *p<* 0.001) in the UK Biobank dataset. However, this association was not significant in the SPECT-China dataset (OR: 1.13 (95% CI: 0.64–1.99); *p* = 0.669). Further adjustment for total cholesterol, triglycerides, and systolic blood pressure did not attenuate these results (**Supplementary Table S3**).

### Bidirectional MR analysis

When analyzing online GWAS datasets using the IVW method, we found that genetically instrumented NAFLD was consistently associated with a higher risk of T2DM (OR: 1.003 (95% CI: 1.002–1.004); *p<* 0.001), but there was no association between genetically instrumented T2DM and NAFLD (ORs ranged from 3.13 to 345.8, all p > 0.05) (**Table 2**). Pleiotropy bias was not detected in these analyses (both *p* > 0.05).

**Table 2:** MR estimates of the causal association between T2DM and NAFLD.

	Effect size (95% CI)	*p*-value
T2DM → NAFLD		
IVW (fixed effects)	28.1 (0.7–1,143.0)	0.08
IVW (multiplicative random effects)	28.1 (0.3–2,386.2)	0.14
MR-Egger	345.8 (0.01–24,295,450.8)	0.31
Weighted median	3.13 (0.01–886.1)	0.69
Test for heterogeneity: *p* = 0.015 (IVW) and *p* = 0.013 (MR-Egger)		
Test for horizontal pleiotropy: MR-Egger intercept = −0.01 (−0.05 to 0.03), *p* = 0.63		
NAFLD→T2DM		
IVW (fixed effects)	1.003 (1.002–1.004)	<0.001
IVW (multiplicative random effects)	1.003 (1.001–1.005)	0.002
MR-Egger	1.01 (1.02–1.00)	0.18
Weighted median	1.003 (1.002–1.005)	<0.001
Test for heterogeneity: *p* = 0.016 (IVW) and *p* = 0.366 (MR-Egger)		
Test for horizontal pleiotropy: MR-Egger intercept = 0.994 (0.990 to 0.998), *p* = 0.224		

The effect size was presented as the odds ratio and its 95% confidence interval.

CI, confidence interval; T2DM, type 2 diabetes mellitus; NAFLD, nonalcoholic fatty liver disease; IVW, the inverse-variance–weighted (IVW) method; MR, Mendelian randomization.

## Discussion

In the observational analysis, we found a significant association between baseline NAFLD and an increased risk of incident T2DM in the SPECT-China and the UK Biobank datasets. The association between baseline T2DM and increased risk of incident NAFLD was observed in the UK Biobank dataset but not in the SPECT-China dataset. Further bidirectional two-sample MR analysis showed consistent evidence that genetically instrumented NAFLD increased T2DM risk while the T2DM→NAFLD relationship was unlikely to be causal.

Over the past decade, several population-based studies have focused on the relationship between NAFLD and T2DM. Almost all of the studies that have used noninvasive imaging techniques (predominantly ultrasonography) to diagnose NAFLD have shown that NAFLD increases the risk of incident T2DM (7–9), echoing our results. However, all of these studies are conducted in the Asian population, mainly South Koreans, and evidence derived from other populations is lacking. Therefore, we conducted the analysis in the UK Biobank cohort of European descent, and similar results were found. Since the diagnosis of NAFLD in the UK Biobank depended on hospital admission records, the prevalence of NAFLD was only 0.98%, which was significantly lower than the average prevalence worldwide. To reduce this selection bias, we further used the FLI to assess the association between NAFLD and incident diabetes, and the results remained unchanged.

On the other hand, several studies point to T2D as a risk factor for NAFLD as well as the progression toward NASH, fibrosis, and HCC (22–24). In our study, the relationship between T2DM and NAFLD was found in the UK Biobank dataset but not in the SPECT-China dataset. This result requires verification in larger cohorts as it may be specific to the Chinese population or just a chance finding due to the small sample size.

However, an observational study is not capable of investigating the causal effect between these two diseases and has many limitations. First of all, the golden standard for diagnosing NAFLD is liver biopsy, which is not feasible in epidemiological studies. Using liver ultrasound and FLI instead may cause diagnostic bias. Moreover, though we carefully adjusted for various confounders, bias from residual and unmeasured confounding may still exist. Therefore, we further conducted bidirectional MR analysis to minimize the effects of confounding factors and elucidate the causal effect between these two diseases, where we found a NAFLD→T2DM relationship. A previous study revealed that genetically predicted higher circulating ALT and AST were related to an increased risk of T2DM (25). Another study found a weak association between genetically instrumented hepatic steatosis and two glycemic traits: fasting glucose and fasting insulin. This study also demonstrated that a one-standard deviation (SD) increase in CT-measured hepatic steatosis led to a 30% increased risk of T2D (26). Since circulating ALT and AST are markers for NAFLD and the glycemic trait is a marker for T2DM, our results are in accordance with their results and provided a more direct supplement.

The NAFLD→T2DM causality is biologically plausible. The key features of NAFLD are hepatic lipid accumulation and hepatic inflammation. In the early period of NAFLD, an elevated hepatic lipid availability combined with the inadequate adaptation of mitochondrial function owing to lipid oxidation could induce the hepatic production of DAG and ceramides, affecting hepatic insulin resistance (27). Moreover, patients with NAFLD have moderate increases in total bile acids (28). Primary bile acids are produced in the liver from cholesterol and then secreted into the intestine as glycine and taurine conjugates. The intestinal microbiota then converts primary bile acids into secondary bile acids, which interact with various nuclear receptors in the intestine such as farnesoid X receptor (FXR) and Takeda G protein-coupled membrane receptor 5 (TGR5) (29, 30). These interactions play an important role in insulin clearance and the regulation of hepatic lipid and glucose metabolism. Insulin clearance is decreased in patients with NAFLD, causing less sensitivity to insulin. By contrast, in the later stages of liver disease, the activation of Toll-like receptor 4 (TLR4) by lipopolysaccharide induces inflammation, ceramide biosynthesis, and insulin resistance (31). With *de novo* ceramide synthesis, ceramides derived from palmitic acid are the most potent at decreasing insulin action and causing insulin resistance (32, 33). These mechanisms worked together in triggering insulin resistance, which is the key pathology of T2DM.

The major strengths of this study include the investigation of the bidirectional association between T2DM and NAFLD among the same Chinese population and validation from a large European cohort. More importantly, Mendelian randomization analysis was further performed to determine the causal effects. There are also several limitations to our study. First, liver biopsy, the current gold standard for diagnosing hepatic steatosis was not feasible in a large epidemiological study. Using a blood marker equation to define liver steatosis may not be accurate enough, and ultrasound has limited sensitivity in detecting minor amounts of fatty infiltration. Second, in the SPECT-China study, OGTT 2-h postprandial glucose was not available to be included in the definition of T2DM. Consequently, some potential patients with T2DM might be misclassified. Third, though we carefully adjusted for various confounders, bias from residual and unmeasured confounding may still exist. Forth, the Mendelian randomization analysis was restricted to volunteers of European ancestry, mostly White British, and the number of SNPs for NAFLD is small. Therefore, whether these findings could generalize to other populations need further research. Moreover, although pleiotropy tests were conducted in our study, there may still be potential SNPs associated with unknown phenotypes.

## Conclusions

In summary, results from both observational analysis and bidirectional MR analysis suggest a potentially causal NAFLD→T2DM relationship. Our findings raise public awareness of the intrinsic link between NAFLD and T2DM and emphasize the intervention strategies that target the prevention of T2DM by active management and treatment of NAFLD.

## Data availability statement

The original contributions presented in the study are included in the article/**Supplementary Materials**. Further inquiries can be directed to the corresponding authors.

## Ethics statement

The studies involving human participants were reviewed and approved by Ethics Committee of Shanghai Ninth People’s Hospital, Shanghai JiaoTong University School of Medicine (approval number 2013(86)) UK National Health Service, National Research Ethics Service North West, National Information Governance Board for Health and Social Care in England and Wales, and Community Health Index Advisory Group in Scotland. The patients/participants provided their written informed consent to participate in this study.

## Author contributions

YL, BW and NW conceptualized this paper. YTY and YFY analyzed the data and wrote the manuscript. All authors contributed to the article and approved the submitted version.
