# Supplementary material for: Nonalcoholic fatty liver disease and type 2 diabetes: an observational and Mendelian randomization study
Source: Front Endocrinol (Lausanne). 2023 May 8;14:1156381. doi: 10.3389/fendo.2023.1156381 (PMC10200946; doi:10.3389/fendo.2023.1156381)
Supplement: Supplementary file 1 [file DataSheet_1.docx]

**Supplemental Figure 1** Flow chart of the study participants.


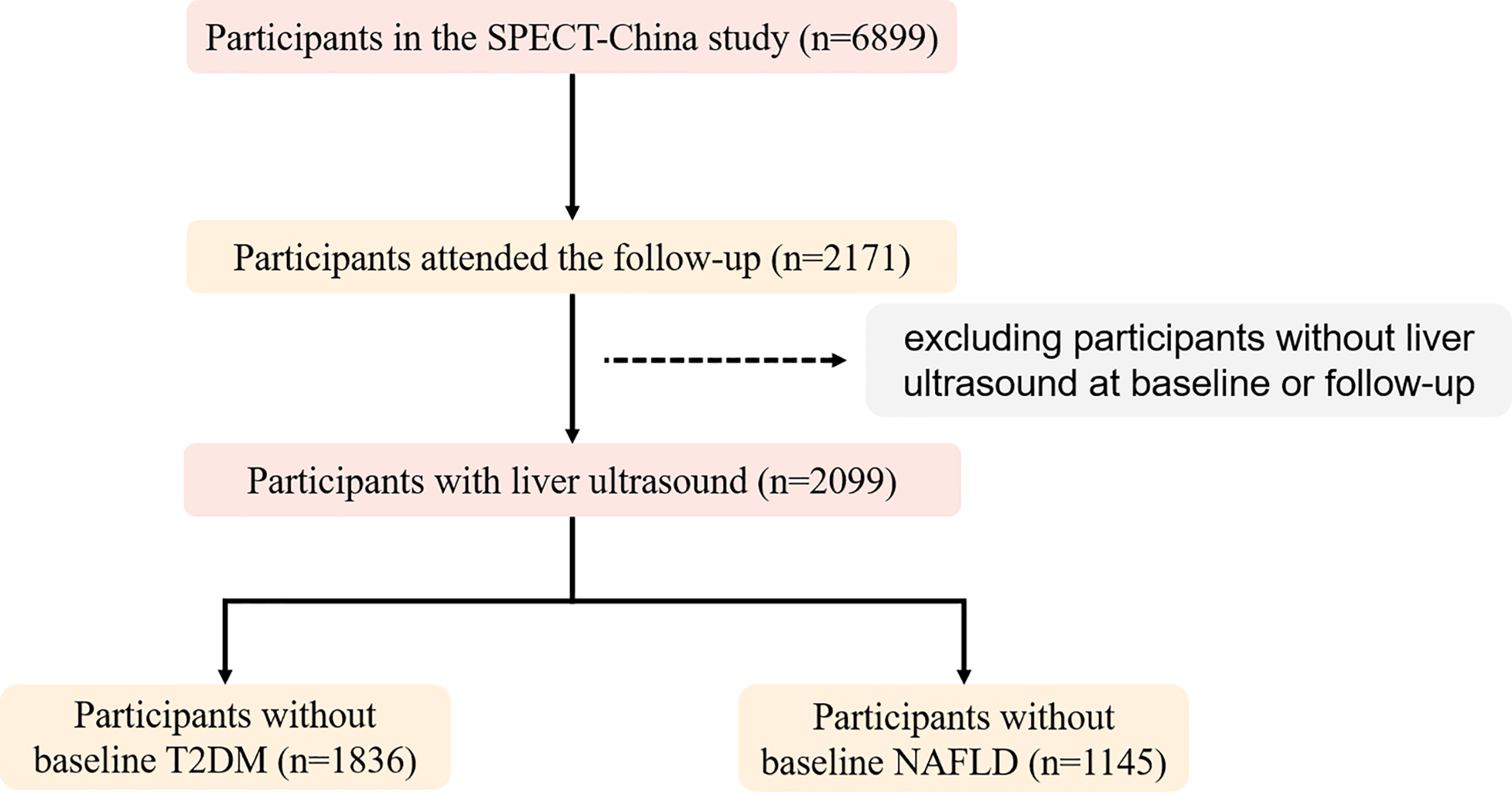


T2DM, type 2 diabetes mellitus. NAFLD, nonalcoholic fatty liver index.

**Supplemental table 1** Information of selected genetic variants associated with T2DM and NAFLD.

| SNP | Chromosome | Position | Effect Allele/other* | EAF, % | Effect size |
| --- | --- | --- | --- | --- | --- |
| **T2DM** |  |  |  |  |  |
| rs4689394 | 4 | 6291003 | G/C | 0.58 | 0.005 |
| rs73077175 | 3 | 49774658 | A/G | 0.43 | 0.012 |
| rs13012438 | 2 | 60584283 | A/C | 0.42 | 0.041 |
| rs11720108 | 3 | 123069058 | C/T | 0.18 | 0.167 |
| rs7633675 | 3 | 185510613 | G/T | 0.31 | 0.007 |
| rs4686471 | 3 | 187740899 | C/T | 0.61 | 0.087 |
| rs11759026 | 6 | 126792095 | A/G | 0.24 | 0.018 |
| rs6885132 | 5 | 14768092 | C/G | 0.12 | 0.136 |
| rs9687832 | 5 | 55861595 | G/A | 0.15 | 0.038 |
| rs10830963 | 11 | 92708710 | C/G | 0.35 | 0.057 |
| rs11763876 | 7 | 7249747 | G/A | 0.15 | 0.089 |
| rs3383 | 6 | 143073041 | T/C | 0.62 | 0.006 |
| rs11658063 | 17 | 36103872 | G/C | 0.70 | 0.012 |
| rs1215468 | 13 | 80707429 | A/G | 0.29 | 0.034 |
| rs111852127 | 16 | 75249170 | A/T | 0.08 | 0.011 |
| rs2303700 | 19 | 7976529 | C/T | 0.67 | 0.009 |
| rs12910361 | 15 | 77782335 | G/A | 0.68 | 0.051 |
| rs34715063 | 15 | 38873115 | C/T | 0.10 | 0.025 |
| rs6567160 | 18 | 57829135 | T/C | 0.18 | 0.148 |
| rs112674299 | 3 | 63982670 | C/T | 0.20 | 0.025 |
| rs4622883 | 3 | 152188290 | A/G | 0.38 | 0.001 |
| rs7376543 | 4 | 49310408 | T/G | 0.76 | 0.047 |
| rs11712037 | 3 | 12344730 | C/G | 0.17 | 0.115 |
| rs9379084 | 6 | 7231843 | A/G | 0.11 | 0.196 |
| rs3798519 | 6 | 50788778 | C/A | 0.21 | 0.044 |
| rs7896600 | 10 | 12255175 | C/G | 0.29 | 0.012 |
| rs1574285 | 9 | 4283137 | G/T | 0.56 | 0.042 |
| rs2793829 | 1 | 120461253 | C/T | 0.14 | 0.041 |
| rs10421566 | 19 | 46159123 | C/T | 0.31 | 0.033 |
| rs10195252 | 2 | 165513091 | T/C | 0.36 | 0.064 |
| rs116782923 | 5 | 102331465 | A/T | 0.06 | 0.077 |
| rs35612982 | 6 | 20682622 | T/C | 0.18 | 0.018 |
| rs231361 | 11 | 2691500 | G/A | 0.29 | 0.010 |
| rs5213 | 11 | 17408404 | T/C | 0.53 | 0.114 |
| rs12611068 | 19 | 13014314 | T/C | 0.54 | 0.027 |
| rs10811660 | 9 | 22134068 | A/G | 0.14 | 0.003 |
| rs1320164 | 8 | 95960767 | G/A | 0.52 | 0.106 |
| rs1496653 | 3 | 23454790 | A/G | 0.31 | 0.003 |
| rs77464186 | 11 | 72460398 | A/C | 0.24 | 0.031 |
| rs4727554 | 7 | 102383690 | C/A | 0.43 | 0.088 |
| rs697239 | 10 | 80947438 | C/T | 0.48 | 0.084 |
| rs860262 | 7 | 28194397 | C/A | 0.49 | 0.011 |
| rs1260326 | 2 | 27730940 | T/C | 0.65 | 0.088 |
| rs992823 | 7 | 15041187 | C/T | 0.53 | 0.016 |
| rs17712208 | 1 | 214150445 | A/T | 0.02 | 0.255 |
| rs1421085 | 16 | 53800954 | C/T | 0.42 | 0.079 |
| rs7903146 | 10 | 114758349 | C/T | 0.20 | 0.020 |
| rs34744311 | 10 | 94467287 | C/T | 0.40 | 0.024 |
| rs1800961 | 20 | 43042364 | T/C | 0.05 | 0.235 |
| rs6679677 | 1 | 114303808 | A/C | 0.15 | 0.064 |
| rs17513135 | 1 | 40035686 | C/T | 0.22 | 0.010 |
| rs77101426 | 2 | 43698753 | G/A | 0.03 | 0.059 |
| rs10274687 | 7 | 156933181 | T/G | 0.32 | 0.032 |
| rs2972144 | 2 | 227101411 | G/A | 0.64 | 0.035 |
| rs3802177 | 8 | 118185025 | G/A | 0.38 | 0.002 |
| rs2796441 | 9 | 84308948 | G/A | 0.42 | 0.053 |
| rs2237895 | 11 | 2857194 | A/C | 0.51 | 0.073 |
| rs9273363 | 6 | 32626272 | A/C | 0.28 | 0.078 |
| rs506770 | 6 | 31785228 | C/G | 0.79 | 0.057 |
| rs2258238 | 12 | 66221060 | T/A | 0.08 | 0.190 |
| rs76895963 | 12 | 4384844 | T/G | 0.03 | 0.361 |
| **NAFLD** |  |  |  |  |  |
| rs72882094 | 2 | 171034900 | G/T | 0.09 | 0.001 |
| rs738408 | 22 | 44324730 | T/C | 0.22 | 0.002 |
| rs73004967 | 19 | 19717056 | G/A | 0.07 | 0.003 |

T2DM, type 2 diabetes mellitus; NAFLD Nonalcoholic fatty liver disease; SNP, single nucleotide polymorphism; EAF, effect allele frequency.

*Effect allele is the risk one associated with the two diseases, and the other is the reference allele.

**Supplemental Table 2** Baseline characteristics of the study population in UK Biobank using FLI to define NAFLD

| Characteristic | Participants without baseline T2DM (n = 488884) | |
| --- | --- | --- |
|  | Incident T2DM (n = 28032) | No incident T2DM (n =434597) |
| Age at recruitment, year | 59.2±7.3 | 56.3±8.1 |
| Sex, % |  |  |
| Female | 41.5 | 55.7 |
| Male | 58.5 | 44.3 |
| Smoking status, % |  |  |
| Never | 44.9 | 55.4 |
| Previous | 41.1 | 34.2 |
| Current | 14 | 10.4 |
| Drinking status, % |  |  |
| Never | 7.8 | 3.9 |
| Previous | 5.9 | 3.2 |
| Current | 86.3 | 92.9 |
| Townsend deprivation index | -0.5±3.4 | -1.4±3.0 |
| Living area, % |  |  |
| Rural | 11.4 | 15.2 |
| Urban | 88.6 | 84.8 |
| College education  or above, % | 21.2 | 33.4 |
| BMI, kg/m^2^ | 31.4±5.6 | 27.0±4.5 |
| Family history of diabetes, % | 24.6 | 13.5 |
| Baseline NAFLD, % | 56.7 | 23.5 |

Continuous variables were expressed as mean ± SD and categorical variables were described as a percentage (%). Characteristics of the study sample were compared by the T test for continuous variables and Pearson Chi square test for categorical variables.

All P<0.05, significantly different from that in the non-progressor group.

FLI, fatty liver index; NAFLD, nonalcoholic fatty liver disease. T2DM, type 2 diabetes mellitus.

**Supplemental Table 3** The association between NAFLD and T2DM in SPECT-China and UK Biobank after further adjustment

|  | Data set | Case (Total participants) | OR/HR 95%CI | P value |
| --- | --- | --- | --- | --- |
| NAFLD to T2DM ^*^ | UK Biobank | 30274 (488884) | 2.17 (1.80-2.62) | <0.001 |
|  | SPECT-China | 129 (1836) | 1.60 (1.03-2.51) | 0.038 |
| T2DM to NAFLD^**^ | UK Biobank | 4896 (501763) | 1.53 (1.35-1.74) | <0.001 |
|  | SPECT-China | 263 (1145) | 1.05 (0.59-1.86) | 0.877 |

*Model was adjusted for age, sex. education level, living area, smoking status, drinking status, economic status, BMI, family history of diabetes, systolic blood pressure, total cholesterol and triglycerides.

** Model was adjusted for age, sex. education level, living area, smoking status, drinking status, economic status, BMI, systolic blood pressure, total cholesterol and triglycerides.

HR, hazard ratio. CI, confidence interval. NAFLD, nonalcoholic fatty liver disease. T2DM, type 2 diabetes mellitus.
